# Sublingual Misoprostol-Induced Rhabdomyolysis and Convulsions in Postpartum Hemorrhage: A Case Report and Literature Review

**DOI:** 10.7759/cureus.59874

**Published:** 2024-05-08

**Authors:** Po-Lu Li, Siou-Ting Lee, Zheng-Xian Lin, Yen-Yue Lin

**Affiliations:** 1 Department of Emergency Medicine, Taoyuan Armed Forces General Hospital, Taoyuan, TWN; 2 Department of Obstetrics and Gynecology, Tri-Service General Hospital, National Defense Medical Center, Taipei, TWN; 3 Department of Obstetrics and Gynecology, Taoyuan Armed Forces General Hospital, Taoyuan, TWN

**Keywords:** postpartum hemorrhage, rhabdomyolysis, convulsion, hyperpyrexia, misoprostol

## Abstract

Postpartum hemorrhage (PPH) remains the leading cause of maternal mortality, primarily attributed to uterine atony. Both the World Health Organization (WHO) and the International Federation of Gynecology and Obstetrics (FIGO) endorse the use of misoprostol not only for the prevention but also for the treatment of PPH. However, the administration of misoprostol is commonly associated with transient pyrexia, attributed to a shift in the hypothalamic set point observed in certain animal studies. Misoprostol-induced hyperpyrexia can occasionally manifest with a prodrome of shivering, particularly when administered via the sublingual route, which achieves a higher and faster maximum plasma concentration compared to vaginal and rectal routes. General management strategies to reduce fever involve removing clothing and blankets, applying cool compresses, administering oral acetaminophen, and ensuring adequate hydration. While some cases have reported misoprostol-induced convulsions, hyperpyrexia leading to convulsions and subsequent rhabdomyolysis is a rare and potentially lethal side effect.

In this case presentation, we emphasize a scenario where misoprostol was employed for the treatment of PPH but led to rhabdomyolysis. Our goal is to highlight the side effects of misoprostol and the significance of considering the initial combination of misoprostol with anti-pyretic management to minimize the risk of hyperthermia-related side effects and prevent additional severe complications.

## Introduction

Postpartum hemorrhage (PPH) remains the most lethal obstetric emergency, with uterine atony being the most common etiology [1,2]. For decades, researchers have been searching for the safest and most effective way to prevent and manage this complication. Medications which have been commonly used include oxytocin, methylergonovine maleate and tranexamic acid. Aside from the above choice, misoprostol is an accepted drug of choice nowadays because of its uterotonic potency [3,4], multiple convenient routes of administration and stability under ambient temperature [5]. However, there are also common side effects associated with postpartum usage of misoprostol such as shivering and pyrexia [6]. Previous studies showed that oral and sublingual use of misoprostol has shown higher rates of shivering and elevated body temperature, which may possibly be because they reach a higher and faster maximum plasma concentration effect than vaginal and rectal routes [7]. However, it is extremely rare that the body temperature ≥ 40.0 ℃ is accompanied by convulsions and leads to rhabdomyolysis.

We report a unique and rare case of severe adverse effects within 2 hours of sublingual misoprostol administered to prevent uterine atony and possible PPH after a successful vaginal delivery, including hyperpyrexia up to 43°C body temperature, generalized convulsions and rhabdomyolysis.

## Case presentation

**Figure 1:**
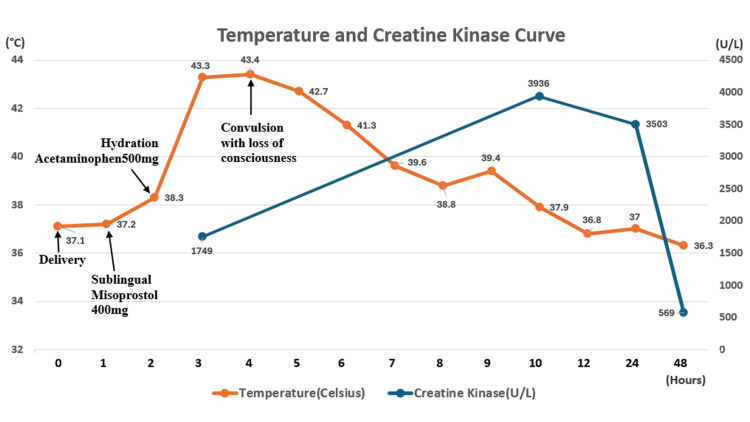
Temperature and Creatine Kinase Curve After sublingual misoprostol, temperature and creatine kinase variations were observed. Immediate administration of acetaminophen for fever, prompt hydration, and cooling were implemented. Data collection started at delivery, with misoprostol administered 0.5 hours post-delivery. Acetaminophen 500 mg was given post-misoprostol and repeated every 4 hours.

A 27-year-old primiparous woman, at 40 weeks and 5 days of gestation, was admitted for labor induction due to overdue status. No abnormalities such as hypertension or proteinuria were detected during prenatal checkups. Labor progressed for approximately 12 hours, culminating in the vaginal delivery of a healthy female infant weighing 3175 g, with Apgar scores of 7 at the first minute and 9 at the fifth minute. While the third stage of labor was completed with the injection of 10 IU of oxytocin, the placenta was delivered and confirmed to be intact. However, poor uterine contraction, accompanied by an estimated blood loss of about 500 ml, was observed. In response, 400 mg of misoprostol was administered sublingually immediately to prevent postpartum hemorrhage. Just 90 minutes later, the patient reported shortness of breath (SpO2 98% on 3 L/minute oxygen via nasal cannula) and exhibited symptoms of high fever (43.4°C) (Figure 1) with shivering chills, hypotension (BP 74/35 mmHg), and tachycardia (HR 137-151 bpm). Aggressive intravenous hydration and an antipyretic agent were administered. In concern of post-partum hemorrhage, the birth canal was evaluated and there was no evidence of active bleeding. This was then followed by an episode of generalized limb convulsion with loss of consciousness. Under the suspicion of eclampsia, we administered lorazepam (2 mg IV) and magnesium sulfate (6 g IV bolus), but the symptoms and hypotension persisted. Given the concern for worsening mental status (E2V2M3), endotracheal tube intubation with ventilator support was done to protect the airway. Other possible differential diagnoses were side effects of misoprostol and amniotic fluid embolism (AFE) because of shortness of breath, hypotension, and systemic involvement. The patient was transferred to intensive care unit (ICU) for further care.

After ICU admission, several examinations were done. Her serum chemistry, including serum creatine, aspartate transaminase (AST)/ Alanine aminotransferase (ALT), electrolyte, thyroid function, blood cell counts, C-reactive protein (CRP) and coagulation profiles, were all within the normal range. Therefore, we excluded sepsis, immune storm, and AFE which is characterized by sudden hypoxia, hypotension, and coagulopathy during labor or within 30 minutes after delivery of the placenta [8].

However, arterial gas with metabolic acidosis and elevated troponin-I level (7424 pg/mL) and Creatine-phospho-kinase (3936 U/L) were found. Electrocardiogram showed only sinus tachycardia. Cardiology was consulted and they felt that this was a response to hypoperfusion. The echocardiogram only showed mild mitral regurgitation. Specifically, there was no hypokinesia, right ventricular (RV) strain pattern or thrombus noted. Urine analysis showed no proteinuria, positive findings for blood (3+) and absence of red blood cells (RBC 0-5). Computed tomography of the head to abdomen, magnetic resonance imaging of the head and electroencephalography all revealed no obvious abnormality. Chest film did not show any acute abnormalities and further bacterial culture reports were negative. The patient was treated with further supportive care, fever subsided about 10 hours later with improved hemodynamic stability without inotropic agent support. After 36 hours of the treatment, the patient was extubated successfully, remaining hemodynamic stable and with no signs of systemic inflammatory response, which help ruling out the initial diagnosis such as eclampsia, AFE, sepsis, maternal cerebrovascular disease, or cardiovascular disease. We then felt that the etiology of the patient's symptoms was a rare reaction to the sublingual misoprostol. The patient was then discharged 5 days later without neurological or obstetric complications.

## Discussion

This was a case of a postpartum woman presented with high fever, sudden onset of hypotension, decreased consciousness, and generalized convulsion. Emergent obstetrics diseases should be considered first, such as eclampsia and amniotic fluid embolism [8, 9]. However, the patient did not present with hypertension or proteinuria and did not respond to the administration of magnesium sulfate, which makes eclampsia less likely. Amniotic fluid embolism is characterized by a triad symptom of hypoxia, hypotension, and coagulopathy during labor or within 30 minutes after placenta delivery [8], which is different from the clinical course of this patient. We evaluated other possible etiologies for postpartum disease processes such as hyperthyroidism, cardiovascular disease, cerebrovascular disease, and infection; however, none of these tests supported these diagnoses. After ruling out these other disease processes, we then concluded her to be the rare complication of sublingual use of misoprostol with the development of extremely high fever with seizure and rhabdomyolysis.

Misoprostol is a synthetic analogue of prostaglandin E1 that was initially employed in the management of peptic ulcers induced by nonsteroidal anti-inflammatory drugs (NSAIDs) [5]. Due to the uterotonic effects, it has been recommended for the treatment and prevention of PPH by the International Federation of Obstetrics and Gynecology (FIGO) and WHO [3, 4]. Although oxytocin is the gold standard drug for the prevention and treatment of PPH, the use of combined misoprostol and oxytocin significantly reduced the amount of blood loss after delivery compared to a higher dose of oxytocin alone [10, 11]. Furthermore, misoprostol offers many advantages as it is heat-stable, cheaper and has variable routes of administration which makes it widely affordable and available, especially in low-resource countries, when compared to oxytocin [7].

According to previous reports, women who received misoprostol postpartum are at risk for shivering and fever [6]. The temperature elevations associated with misoprostol are due to hypothalamus adjustment [12]. E-series prostaglandins (PGEs) play an important role in the endogenous fever mechanism, especially prostaglandin E2 (PGE2) which is known as the mediator of fever induction, through interaction with the EP3 receptor, which is one of the subtypes of PGE receptors [13, 14]. Currently, there is no evidence that prostaglandin E1 (PGE1), of which misoprostol is an analogue, acts differently from PGE2. Furthermore, the biologically active form of misoprostol, misoprostol acid, is thought to bind to EP3 receptors as PGE2 does [15]. As a result, in these cases with fever, we theorize that misoprostol may mimic endogenous PGEs in the thermoregulatory pathway by shifting the hypothalamic set points upward and causing fever.

Generally, a total daily dose of misoprostol 1600 μg is usually tolerated with only mild gastrointestinal discomfort [16]. Diarrhea is the main adverse effect, usually mild and self-limited, that resolves in 2-6 hours. Hyperpyrexia sometimes occurs with the prodrome of shivering, and is associated with sublingual routes of administration [17], which achieve a higher and faster maximum plasma concentration than vaginal and rectal routes [7]. General management for reducing fever includes removing clothes and blankets from the patient, applying cool compresses, administering oral acetaminophen and adequate hydration. In addition, misoprostol-induced fever will follow a predictable pattern [7]. There is a sharp increase within the first hour of misoprostol usage, a peaked temperature in 1-2 hours, and a gradual decline over 3 hours [7, 18]. Rarely, fever above 40°C, but the average remains less than 2 hours and measured below 38°C in 6 hours [18].

Some prospective observational studies conducted in Ecuador and Argentina [7, 18], that enrolled women with atonic PPH receiving misoprostol, reported that 75.5% experienced transient shivering and fever. However, instances of high fever above 40°C were rare, approximately 12.2% in some population groups. Using multivariate logistic regressions, they explored potential predictors of developing fever ≥ 38.0°C and high fever ≥ 40.0°C after treatment with sublingual misoprostol (800 mcg) and found that pre-delivery hemoglobin < 11.0g/dl, rapid placental expulsion, and higher age of the woman were significant predictors of misoprostol-induced high fever≥ 40.0°C.

In our case, a 27-year-old woman with a pre-delivery hemoglobin level of 10.6 g/dL experienced placental expulsion within ≤5 minutes after the birth of the baby. However, she not only developed a fever exceeding 43°C but also experienced a prolonged duration of approximately 10 hours before gradually subsiding (Figure 1). Severe complications, such as convulsions and rhabdomyolysis, also manifested and were evident in laboratory results showing elevated creatine kinase (CK) levels and tea-colored urine. To our knowledge, this adverse effect is very rare and not well described.

Although eclampsia is the primary consideration in postpartum seizure, when the clinical scenario involves convulsion followed by hyperpyrexia after using misoprostol, we should consider the side effects of misoprostol use. The recommended treatment includes aggressive hydration, cooling, and the use of antipyretic agents, rather than magnesium sulfate. Postpartum convulsions can be challenging, and the underlying cause can be more than eclampsia. It's crucial to bear in mind the side effects of misoprostol use and promptly manage them to prevent critical complications. Considering preventive hydration and the use of antipyretic agents, particularly acetaminophen, after misoprostol administration in the future, especially for those with predictors of misoprostol-induced high fever (such as pre-delivery hemoglobin < 11.0g/dl, rapid placental expulsion, and higher age of the woman [18]), may help alleviate hyperpyrexia and the development of serious complications, such as rhabdomyolysis.

## Conclusions

PPH is a universal and unpredictable occurrence. Effectively managing PPH is critical, impacting not only obstetricians but also emergency physicians. Misoprostol is employed as part of the treatment protocol. Our aim is to highlight the risk of rhabdomyolysis, a severe side effect associated with misoprostol, and advocate for the early incorporation of misoprostol into the treatment plan. This integration, coupled with antipyretic management, is proactive in preventing hyperthermia and potentially life-threatening complications.
